# National digital strategies and innovative eHealth policies concerning older adults’ dignity: a document analysis in three Scandinavian countries

**DOI:** 10.1186/s12913-023-09867-w

**Published:** 2023-08-10

**Authors:** Moonika Raja, Ingjerd G. Kymre, Jorunn Bjerkan, Kathleen T. Galvin, Lisbeth Uhrenfeldt

**Affiliations:** 1 Faculty of Nursing and Health Sciences, Nord University, Bodø, Norway; 2 Faculty of Nursing and Health Sciences, Nord University, Levanger, Norway; 3 School of Sport and Health Sciences, University of Brighton, Brighton, UK; 4 Ortopedic Surgery, Kolding Hospital, Kolding, Denmark; 5 Institute of Regional Health Research, Southern Danish University, Odense, Denmark

**Keywords:** Digital strategies, Dignity, Document analysis, eHealth, Older adults, Qualitative

## Abstract

**Background:**

Scandinavian countries are internationally recognised for leading the way in older adult care and in digitally transforming healthcare. Dignity has become a central value in care for older adults in all three Scandinavian countries. Investigating documents about digitalisation in these countries can offer insights into how the dignity of older adults is impacted by digitally transforming healthcare. This study aims to provide knowledge about digital strategies and eHealth policies concerning older adults’ dignity in three Scandinavian countries: Norway, Sweden and Denmark.

**Methods:**

National-level documents by the Norwegian Directorate of eHealth, the Norwegian Directorate of Health, the Swedish Ministry of Health and Social Affairs and the Danish Ministry of Health concerning older adults were used as data sources. In addition, a systematic search of databases, informed by the Joanna Briggs Institute framework for systematic reviews of text and opinion papers, was undertaken to find relevant papers. All extracts concerning national digital strategies or innovative eHealth policies were deductively coded. Thereafter, extracts concerning older adults were inductively coded using a thematic analytic approach.

**Results:**

A total of 26 sources satisfied the inclusion criteria, 14 governmental papers and 12 other papers. The three countries’ national digital strategies focused on access to digital technologies and continuous learning for digital skills. The included papers describing national eHealth policies underlined the importance of placing the patient at the centre of healthcare and how digital systems can increase feelings of safety. Both types of documents concerned access to data, digital device security and the human dimension of care.

**Conclusion:**

The findings present evidence on Scandinavian countries’ national digital strategies and innovative eHealth policies concerning older adults’ dignity. The documents describe a lack of digital competence among older adults, resulting disengagement may put their well-being and human dignity at risk. Findings also underline the importance of security and at the same time the human dimension of care: Use of new digital systems must be meaningfully integrated into digital strategies and eHealth policies. All three Scandinavian countries strategies and policies underline the importance of equal access to healthcare services, as thus they promote a stance of dignified care.

## Background

The European Union`s Charter of Fundamental Rights [1] places dignity as the foundation of all human rights. The term “dignity” comes from the Latin word *dignitas* and refers to values associated with being human, such as worthiness and honour [2]. Dignity is closely linked to an understanding of what makes a person feel human [3]. It is inviolable and must be protected and respected [1]. Dignity has many possible variations and nuances that human beings refer to in a meaningful way [4]. It is an affirmation that can be ruptured or lost through vulnerability [4]. When dignity is taken away, it would diminish one`s personhood [5]. Dignity is referred to as a core value underlying medical practice and as a subjective experience which is related to autonomy and identity [3, 6]. The European Commission underlines the importance of delivering healthcare innovations in a dignified way [7].

Scandinavian countries (Norway, Sweden, and Denmark) are internationally recognised as digital frontrunners in the European and even global contexts [8, 9]. In Scandinavian countries, an important aim of policies relating to ageing is for people to remain in good physical and mental health for as long as possible [10]. Digital technologies have been part of the solution by providing sustainable care, but at the same time, the age-based digital divide has led to inequality for older adults around the world [11].

Welfare models in Scandinavian countries are based on citizens’ high levels of education and long life expectancies, combined with investments in innovation and research [10]. Scandinavian welfare states can be characterised as providing high-quality services for all age groups, as regional and municipal authorities play a central role in the delivery of key services [12]. Healthcare in Scandinavian countries, as part of the Nordic welfare model, is underpinned by the basic values of compassion, tolerance and the conviction of equality [13]. Healthcare systems in Scandinavian countries are tax-funded. National, regional and municipal governments are responsible for the provision of care and may contract public and private providers [10].

Digital technologies are viewed in Scandinavian countries as tools to fulfil existing national healthcare responsibilities and realise local and regional goals [8]. All three Scandinavian countries have strong underlying digital strategies that support their digital healthcare policies and innovative plans [14–16]. The Norwegian Government follows strategies to modernise, simplify and improve the public sector by using the opportunities that digitisation offers, and the main priorities are affected by international trends [14]. The Swedish Agency for Digital Government implements digital policies for their digital services to be based on users’ needs, include digital identities and e-invoicing, and be accessible to everyone [15]. The Danish Agency for Digitalisation implements digital policies to use digital-ready legislation to support and benefit citizens in digital society and ensure that personal data is handled safely [16].

Scandinavian countries have a strong history of delivering digital health solutions that support and optimise their national healthcare systems [17]. Denmark has been at the forefront of the integration of digital healthcare for 20 years [18]. As early as 2010, the New York Times claimed that Denmark was leading the way in digital care [19]; the transformation has continued, and a decade later, a United Nations (UN) survey ranked Denmark first out of 193 Member States in terms of digital government [9].

Although there are geographical differences between the Scandinavian countries, there are many similarities between their digital healthcare strategies [17]. The Norwegian digitalisation strategy in healthcare between 2017 and 2022 has a vision of all citizens enjoying easy and safe access to healthcare services, including digitalised patient records available through a public eHealth portal, access to e-consultations and e-prescriptions, easy scheduling of doctor’s appointments, and information about available healthcare services [20]. Sweden’s digital healthcare strategy for 2025 is for all citizens to achieve good and equal healthcare, and to strengthen their own resources for participation in social life [21]. The Danish Digital Health Strategy between 2018 and 2022 is for citizens to experience the healthcare system as a coherent network based on a citizen-centric approach, with a focus on digitalisation and the use of health data in the context of direct treatment, care and prevention [22].

European countries provide one of the most distinctive examples of demographic ageing, and population projections suggest that the pace of ageing of Europe will quicken in the coming decades [23]. Today, over one in nine people in Norway are aged 70 years or older. The prognosis, based on medium fertility, life expectancy and net migration, is that roughly every fifth person in Norway will be over 70 years old by 2060 [24]. Sweden and Denmark also expect the largest demographic population increase to be among older adults [25, 26]. In recent decades, population ageing has been one of the main demographic trends in the Scandinavian countries and this trend is projected to continue [10].

Population ageing is rapidly transforming society [23]. As it is a major development, how to respond to population ageing has emerged as a central question in public debate and on policy agenda [10]. An increasing number of older adults entails challenges for social policy and healthcare systems. Modern assistive technologies, in particular digital technologies, are being heralded as part of the solution to providing sustainable care. Countries are developing new digital goods and services that can be adapted to the needs of older adults [27–29].

Digital technologies can help provide sustainable care, but unfamiliar systems may impact older adults’ dignity. From a phenomenological point of view, human dignity is the affirmation of something valuable in oneself or another and can be ruptured [4]; therefore, the challenge of keeping up with technological shifts may make older adults vulnerable and affect their dignity [30]. However, dignity has become a central value in care for older adults in all three Scandinavian countries [31–34]. Preserving human dignity in the demanding situation of digital innovations is challenging and involves facing issues such as dependence, privacy, vulnerability and the need to be treated as an individual [34]. Therefore, as digital innovations can impact older adults` dignity, attention should be directed to their potential for the delivery of dignified care [7]. In January 2011 a new policy to support older adults’ rights to dignified care and well-being, “The guarantee of dignity”, passed into legislation in Norway and Sweden [32–34]. The aim of the regulation is to ensure that care for older adults, whether home-based or institutional, is organised in a way that contributes to dignified, meaningful and secure ageing. In the Norwegian strategy, dignified care is interpreted as keeping a person safe and having meaning in their old age. Having living arrangements based on one’s needs allows one to retain the ability to function in daily life [35]. In the Swedish strategy, dignified care underlines the importance of personal integrity, self-determination, participation and individualised care [36]. In the Danish strategy, the dignified care of older adults focuses on involving and empowering every citizen, according to their individual needs, to maintain their independence and gain control of their own life [37]. All three countries’ strategies underline the importance of individualised dignified care for older people [35–37].

Documents, through a systematic approach, can help researchers uncover meaning, develop understanding, and discover insights relevant to the research problem. They also provide background and context and serve as a means of tracking change and development [38]. A preliminary search of PubMed, CINAHL and Scopus gave few results about earlier research in Scandinavian countries concerning policy documents for healthcare systems. Some examples are by researchers Frennert, Triantafillou and Dahlborg with collegues [39–42]. However, these studies did not explore digital strategies and eHealth policy concerning the dignity of older adults. While a recent Scandinavian study investigated how the concept of “a patient” is constructed in central policy texts in these countries [39], it did not address eHealth, dignity or older adults.

## Aim

The aim of this study is to provide knowledge about digital strategies and eHealth policies concerning older adults’ dignity in three Scandinavian countries: Norway, Sweden and Denmark. This study is guided by three research questions:

- 1) Which digital strategies concerning older adults are described in documents, including those by the Norwegian Directorate of eHealth, the Norwegian Directorate of Health, the Swedish Ministry of Health and Social Affairs, and the Danish Ministry of Health ?
- 2) Which eHealth policies concerning older adults are described in documents, including those by the Norwegian Directorate of eHealth, the Norwegian Directorate of Health, the Swedish Ministry of Health and Social Affairs, and the Danish Ministry of Health?
- 3) Which national strategies for digital development and eHealth have innovative power in relation to the dignity of older adults?

## Methods

In this qualitative study the core values of dignity and a subjective experience of autonomy and identity are central to framework and lie behind our deductive analysis of how healthcare innovation is led by healthcare strategies and policies [3, 6]. Documents were gathered as a data source to discover insights guided by the research questions [38, 43]. In line with O’Leary [43], the document analysis process comprised the following steps: (a) planning; (b) gathering; (c) reviewing; (d) interrogating; (e) reflecting; and (f) analysing data. In the analysis, the data was first deductively coded, following Bowen [38], and thereafter extracts concerning older adults were inductively coded using a thematic analytic approach following Braun and Clarke [44].

### Planning, data gathering and reviewing

The criteria for inclusion in the study was textual and opinion papers exploring national digital strategies and eHealth policies concerning older adults in Norway, Sweden or Denmark. National documents by the Norwegian Directorate of eHealth, the Norwegian Directorate of Health, the Swedish Ministry of Health and Social Affairs and the Danish Ministry of Health concerning older adults were used as data sources. Government reports, expert opinions, discussion papers and position papers published in Danish, English, Norwegian and Swedish were considered. In addition, a systematic search guided by the Joanna Briggs Institute (JBI) framework for systematic reviews of textual and opinion papers in databases was undertaken to find relevant papers [44]. According to JBI framework [45], reports from professional organizations, consensus guidelines, expert consensus, policy reviews, papers about case reports and studies including expert opinion were included. We began searching in 2021 and papers published from January 2016 were considered for inclusion, as the World Health Organisation (WHO) considers topical updates from the last five years about countries that have a comprehensive national health sector policy with goals and targets [46].

To find governmental papers, a systematic search was conducted on the websites of the Norwegian Directorate of eHealth, the Norwegian Directorate of Health, the Swedish Ministry of Health and Social Affairs and the Danish Ministry of Health. We used keywords in English and in relevant Scandinavian languages. To find other documents, a controlled vocabulary and keyword search was conducted using the following medical and social science electronic databases: CINAHL, MEDLINE via PubMed, ORIA and Google Scholar. The search strategies were drafted by the researchers in collaboration with a university librarian. The keywords used during the search are shown in Table 1. We used the main keywords throughout. Boolean logic containing combinations of MeSH Terms and Text Words was used [47].

**Table 1:** Keywords used during the search

Keywords	Digital	Arrangements	Danish
	Digitalization	Methods	Denmark
	eHealth	Policies	Nordic
	Electronic health	Policy	Norwegian
	Health informatics	Strategies	Norway
	mHealth	Strategy	Scandinavia
	Network Assistive technology	Systems	Scandinavian
	Technology		Sweden
	Telehealth		Swedish
	Telemedicine		
MeSH terms	Digital technology	Methods	Denmark
(MEDLINE)	Technology	Policy	Norway
	Telemedicine		Scandinavian and Nordic Countries
			Sweden
Headings	Assistive Technology	Health Policy	Denmark
(CINAHL)	Digital technology	Public Policy	Norway
	Health Informatics		Scandinavia
	Health Information Networks		Sweden
	Telehealth		

In search strategy for databases, we used only English search terms, but in searches on governmental websites were also included terms in Norwegian, Swedish and Danish. The specific terms changed slightly depending on the database and website. The final search reports were exported into Rayyan [48]. After removing duplicates, all governmental papers were screened by two authors (MR and IGK) and the other texts were screened by two authors (MR and LU). Papers were included in the study according to the inclusion and exclusion criteria shown in Table 2. The reference lists of potential papers were visually scanned.

**Table 2:** Inclusion and exclusion criteria [45]

	Include	Exclude
Phenomena of interest	Publications that describe digital strategies and eHealth policies provided by national healthcare systems	Publications that do not describe digital strategies and eHealth policies provided by national healthcare systems
Context	About Norway, Sweden, Denmark, Scandinavian countries	Provide no separate information about Norway, Sweden, Denmark or Scandinavian countries
Types of publications	Government reports, expert opinion, discussion papers, reports from professional organizations, policy reviews, academic papers about case reports and studies including expert opinion	Statistical reports, epidemiological reports, other academic papers (not about case reports and studies including expert opinion)
Language	English, Norwegian, Swedish or Danish	Not in English, Norwegian, Swedish or Danish
Types of outcomes	Digital strategies and eHealth policies that impact older adults	Not about digital strategies and eHealth policies that impact older adults
Period	Published January 2016 and after	Published before January 2016

Included texts were reviewed critically using the JBI Critical Appraisal Checklist for Text and Opinion Papers [49]. The checklist included six questions concerning the source of the paper, the field of expertise, the focus and logic of the opinion, and references to extant literature. Each question was answered on a scale of Yes, No, Unclear or Not Applicable. Papers that received a “Yes” to 4 or more questions were included in the study.

### Data interrogating, reflection and analysis

In accordance with O’Leary [43], background information on author, year, purpose and style was extracted. Pertinent information from data sources was identified and separated from that which was not pertinent [38]. Information that did not concern national digital strategies and eHealth policies for older adults, e.g. information about babies, childcare or private companies, was not considered pertinent and was not extracted. First, all pertinent extracts were deductively coded to distinguish between national digital strategies and innovative eHealth policies, according to the devised framework [38]. Then, under these two categories, inductive analysis was undertaken using a thematic analytic approach, with the themes capturing significant aspects of the data concerning research questions [44]. Each step of the analysis is illustrated in Table 3. Any discrepancies in the initial coding were discussed among the researchers until a consensus was reached. The analysis involved constantly moving back and forth between the entire data set, the coded extracts of the data, and the analysis of the data that emerged [50]. The final codes were subsequently categorised according to research questions into the three overarching categories: (a) national digital strategies; (b) innovative eHealth policies; and (c) digital strategies and eHealth policies concerning older adults’ dignity. These three categories were then organised into themes [44]. Descriptions of these are presented in the results section.

**Table 3:** A three-step movement from included documents to the final themes

**Material from the documents**:
Digital solutions must be easy-to-use, quick and ensure high quality. A user friendly and simple digital public sector and better use of data [51]. Digital safety and security of businesses are essential to being able to exploit the opportunities offered by digitalisation [51] For many patients and types of examinations it is not relevant to replace physical meetings with digital solutions [52] Training programs for older users to master technological tools lead to additional benefits [29] Therefore, it must be possible for digitisation to support those who can cope with and want a digitised health system, while simultaneously allotting time for patients, including at-risk elderly citizens, with a greater need for face-to-face interaction [22] Cooperation with the private sector on digitalisation will be enhanced [53] Other ethical issues in eHealth and elderly users are related to the potential replacing of offline services and personal face-to-face contact [54] Assistive technologies can lead to gains in independence through human-non-human contact, but this in turn can have negative effects on the levels of social inclusion and human interaction [29]

**Step 1: Separating pertinent information from data sources**
**Pertinent information from data sources:**
Digital solutions must be easy-to-use, quick and ensure high quality. A user friendly and simple digital public sector and better use of data [51] For many patients and types of examinations it is not relevant to replace physical meetings with digital solutions [52] Training programs for older users to master technological tools lead to additional benefits [29] Therefore, it must be possible for digitisation to support those who can cope with and want a digitised health system, while simultaneously allotting time for patients, including at-risk elderly citizens, with a greater need for face-to-face interaction [22] Other ethical issues in eHealth and elderly users are related to the potential replacing of offline services and personal face-to-face contact [54] Assistive technologies can lead to gains in independence through human-non-human contact, but this in turn can have negative effects on the levels of social inclusion and human interaction [29]

**Step 2: Deductive coding**
**Category digital strategies:**
Digital solutions must be easy-to-use, quick and ensure high quality. A user friendly and simple digital public sector and better use of data [51] Training programs for older users to master technological tools lead to additional benefits [29]
**Category eHealth policies:**
For many patients and many types of examinations, it is less relevant to replace physical meetings with digital solutions [52] Therefore, it must be possible for digitisation to support those who can cope with and want a digitised health system, while simultaneously allotting time for patients, including at-risk elderly citizens, with a greater need for face-to-face interaction [22] Other ethical issues in eHealth and elderly users are related to the potential replacing of offline services and personal face-to-face contact [54] Assistive technologies can lead to gains in independence through human-non-human contact, but this in turn can have negative effects on the levels of social inclusion and human interaction [29]

**Step 3: Inductive coding**
**Final theme in the results:**
From these extracts under category eHealth policies (together with other relevant extracts) immerged theme “Access to data and the human dimension of care”. Digital solutions are not always the best, especially if they risk replacing all face-to-face contacts with digital solutions, this can have negative effects on levels of social inclusion and human interaction. Reduced social stimulus could lead to person`s need for human contact not be met, and thereby affect human dignity in a negative way

## Results

**Fig. 1:**
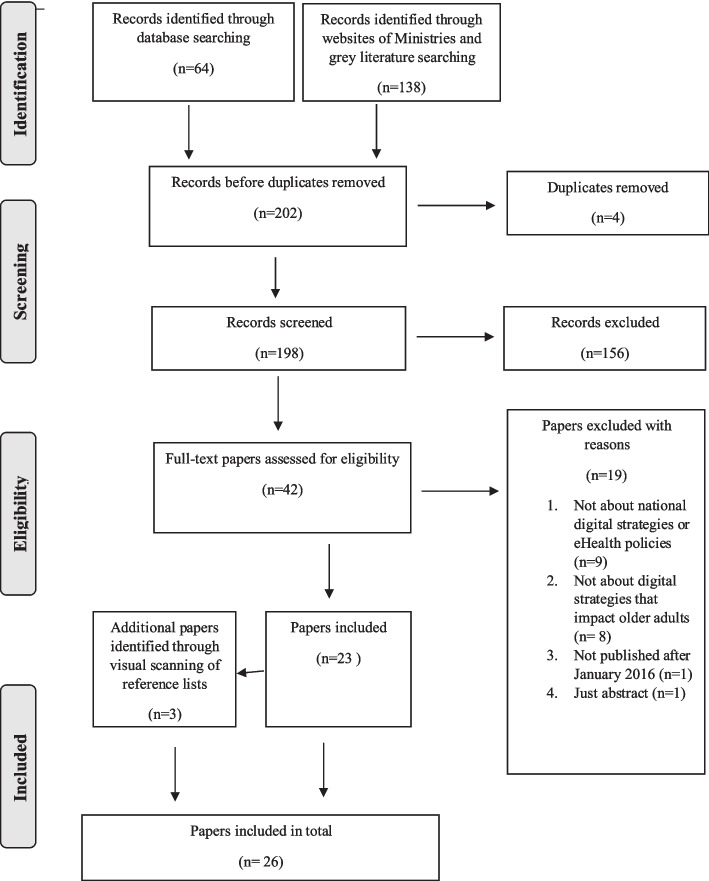
PRISMA flow diagram illustrating the search strategy. This flow diagram provides the phases of paper identification and selection, which resulted in the identification of 26 papers that were deemed eligible for inclusion in the study. Prepared in accordance with Tricco et al. [55]

In accordance with the inclusion criteria (Table 2), papers describing digital strategies and eHealth policies provided by national healthcare systems that impact older adults in three Scandinavian countries — a total of 26 documents were included (see Fig. 1). Of these, 8 focus on Sweden, 6 on Norway, 5 on Denmark, 1 on Norway and Sweden and 6 on all three Scandinavian countries.

Of the 26 sources, 17 described strategies and policies that influence older adults [8, 17, 21, 22, 51–53, 56–65] and 9 focused directly on older adults [29, 37, 40, 41, 54, 66–69]. Background information on the author(s), year, style, country and purpose of each paper can be found in Table 4. Documents describing national digital strategies focused on access to digital technologies and continuous learning for digital skills. Documents describing national eHealth policies underlined the importance of the patient at the centre of healthcare and the feelings of safety that digital systems can provide. Both types of documents were concerned with digital device security, access to data, and the human dimension of relationships and care. The results below answer the three research questions with thematic findings on: a) national digital strategies concerning older adults, b) national eHealth policies concerning older adults and c) digital strategies and eHealth policies concerning older adults’ dignity.

**Table 4:** Background information on authors, year, style and purpose of the paper

	**Governmental Documents**				
	**Author(s) and country**	**Year**	**Purpose of the paper**	**Style**	**About**
1	The Government/ Local Government Denmark/ Danish Regions; Denmark [51]	2016	To present the digital strategy 2016–2020	Publication for public	Digital strategies
2	Ministry of Health and Social Affairs; Sweden [21]	2016	To present the Swedish common vision for eHealth 2025	Publication for public	eHealth policies
3	Ministry of Social Affairs; Sweden [66]	2017	To present the national quality plan for older people’s care	Policy document	eHealth policies
4	Danish Ministry of Health; Denmark [22]	2018	To present the digital health strategy 2018–2022	Publication for public	eHealth policies
5	Norwegian Ministry of Health and Care Services; Norway [67]	2018	To present the quality reform for older persons: *Live Your Whole Life*	Publication for public	eHealth policies
6	Healthcare Denmark, Danish Ministry of Health; Denmark [37]	2019	To present the Danish approach to coherent care of older people and solutions that help to improve quality of life for older citizens	Publication for public	eHealth policies
7	Norwegian Ministry of Health and Care Services; Norway [52]	2019	To present a national health and hospital plan	Policy document	eHealth policies
8	Norwegian Center for E-health Research; Norway [69]	2019	To report outcomes of the national project *Social, digital contact to mobilise against loneliness among older people*	Report	Digital strategies
9	National Board of Health and Welfare; Sweden [56]	2019	To present a strategic plan to support good care that is close to patients	Policy document	eHealth policies
10	Ministry of Local Government and Modernisation; Norway [53]	2019	To present the digital strategy for the public sector 2019–2025	Publication for public	Digital strategies
11	Norwegian Ministry of Health and Care Services; Norway [57]	2020	To present a summary of the national health and hospital plan	Summary of policy document	eHealth policies
12	Ministry of Enterprise and Innovation; Sweden [58]	2020	To present Sweden`s national life science strategy	Publication for public	eHealth strategies
13	Norwegian Directorate of Health; Norway [59]	2021	To present overview and new knowledge from the national welfare technology program	Report	eHealth policies
14	Danish Ministry of Health, Healthcare Denmark; Denmark [60]	2021	To present the Danish super-hospital programme	Publication for public	eHealth policies
	**Other Documents**				
	**Author(s) and country(ies) represented**	**Year**	**Purpose of the paper**	**Style**	**About**
1	Larsen, Sørensen, Petersen and Kjeldsen, Denmark [61]	2016	To present results from a multi-stakeholder project that developed a new concept, a “shared service centre” for telemedicine that is envisioned as working across different telemedical initiatives to support the implementation and wider adoption of telemedicine	Article	eHealth policies
2	Essen, Scandurra, Humphrey, Johansen, Kierkegaard, Koskinen, Liaw, Odeh, Ross, Ancker, Norway, Sweden and Denmark [62]	2018	To compare patient-accessible electronic health records policy and services in ten countries	Article	eHealth policies
3	Nordic Innovation, Norway, Sweden and Denmark [17]	2018	To provide information about Nordic Countries` digitally-led healthcare	Publication for public	eHealth policies
4	Randall, Berlina, Teräs and Rinne; Nordregio, Norway, Sweden and Denmark [63]	2018	To report the preliminary findings of a literature and policy review that was conducted as part of the in-depth study: *Digitalization as a tool for sustainable Nordic regional development*	Report	Digital strategies
5	Hägglund, DesRoches, Petersen and Scandurra, Sweden [64]	2019	To bring attention to patients’ limited access to health records	Editorial	eHealth policies
6	Limen, Hill-Cawthrone, Niezen and Tennøe; European Parliamentary Technology Assessment, Norway and Sweden [29]	2019	To provide an up-to-date international overview of policies linked to the topic of technologies in older people’s care	Report	Digital strategies and eHealth policies
7	Randall and Berlina; Nordregio, Norway, Sweden and Denmark [8]	2019	To summarise the work and results achieved within the study on digitalisation titled *Governing the digital transition in Nordic regions: The human element*	Report	Digital strategies
8	Frennert, Sweden [40]	2019	To illustrate how technological change and municipal employment of welfare technologies is employed in Sweden	Article	eHealth policies
9	Schliemann, Danielsen, Virtanen, Vuokko, Hardardottir, Alsaker, Aksnes, Ekløf and Ericsson, Norway, Sweden and Denmark [65]	2019	To summarise the discussions that took place in a seminar in Copenhagen in late 2018 on eHealth standardisation in Nordic countries	Report	eHealth policies
10	Frennert, Sweden [41]	2020	To explore how welfare technologies are implemented in Swedish practices of caring for older people	Article	eHealth policies
11	Stjernberg, Sigurjonsdottir, Meijer; Nordregio, Norway, Sweden, Denmark [68]	2021	To examine policies and initiatives to promote the *silver economy* and the closely related concepts of healthy ageing, active ageing and age-friendliness	Report	Digital strategies and eHealth policies
12	Valokivi, Carlo, Kvist and Outila, Sweden [54]	2021	To analyse policy documents	Article	eHealth policies

### National digital strategies concerning older adults

Documents about national digital strategies concerning older adults in the three Scandinavian countries focused on user access to digital technologies and continuous learning for digital skills.

#### Access to digital technologies

All three Scandinavian countries have adopted digital strategies and emphasise the importance of equal access to digital technologies [8, 21, 29, 63]. At the same time, it is essential to acknowledge that access to digital technologies is not equal. “There is a need for equal access to digital technologies” [63, p. 7]. In the context of the high rates of usage of digital technology in Scandinavian countries, taking services online has sometimes been seen as synonymous, to an extent, with making services more accessible to all citizens, but this is not, in fact, the case [8]. In Scandinavian countries, there is a digital divide related to socio-economic status, age, gender, and health, as well as disparities between urban and rural areas concerning the availability of digital infrastructure and the adoption of digital technologies [8, 63]. Several documents indicate that a large proportion of citizens lack the infrastructure and skills required for full participation in digital life. There is still great potential for older people to benefit from digital technologies, as the three Scandinavian countries are among the top-ranked in Europe in the rate of older people who report having above-basic overall digital skills [53, 68]. For instance, while more than 40% of the EU population aged 65–74 had never used a computer according to survey results in 2017, the corresponding rates were only 5% in Norway and Sweden and 11% in Denmark [68]. A lack of digital technology skills and knowledge in rural areas limits the possibilities for innovative service provision and customer use [40, 58, 64, 67].

#### Continuous learning for digital skills

The development of innovative digital solutions that support demographic challenges is advanced in Scandinavian countries and requires continuous learning to keep up [8, 9, 42, 57]. Documents included suggest that programmes aiming to support and increase digital competence can help older adults adapt better to the digital environment [21]. **“**Digital solutions must be easy-to-use, quick and ensure high quality” [42, p. 14]. Citizens should be equipped to operate in the digital environment [9, 17, 41, 51, 64]. In reports on European countries, including Scandinavian countries, it becomes obvious that older adults’ participation in society requires far more than just a simple technological fix [29]. Digital strategies in the three Scandinavian countries include programmes aiming to support and increase the digital competence of older adults [29, 41, 56, 69]. Included documents indicate that training programmes which enable older adults to master technological tools can provide additional benefits, such as larger social networks and reduced loneliness [29, 69].

### National eHealth policies concerning older adults

Documents about national eHealth policies concerning older adults in the three Scandinavian countries underline the importance of the patient at the centre of healthcare and the aspect of how digital systems can increase feelings of safety.

#### Patients at the centre of healthcare

The aim of digitalisation is to provide patients more opportunities to participate more fully in their own healthcare, seeking to put the patient at the centre of care and engaging them as equal stakeholders within the care continuum [17, 52, 56, 57]. This is intended to bring healthcare providers and patients closer together. The voice of patients is important in interactions with healthcare providers, but also in the development of eHealth systems [17, 29, 53, 54, 67]. “It is obvious to ask the question: “What is important to you?” when decisions must be made. The patient is an active participant” [52, p. 18]. The documents we surveyed emphasise that strategies for the implementation of technologies should address the individual’s conditions and needs and how eHealth can meet those particular needs [29, 66, 67]. eHealth solutions can help to maintain older adults’ quality of life, integrity, independence and mastery [29, 58, 67]. Digitalisation enables more tasks to be performed close to patients, looking at each person as a whole, not just at their individual diagnoses [8, 22, 52].

#### Digital systems increase feelings of safety

Digital systems crucially help provide feelings of safety [22, 29, 52, 56, 59]. User-friendly care technologies, including safety alarms, electronic door locks, remote health monitoring, GPS monitoring and mobile applications, can offer support in different ways. GPS monitoring can prevent older adults from getting hurt if they get lost, and GPS alarms allow individuals with dementia to decide where and when they want to take walks outside [29, 56]. Monitoring can also provide a sense of peace and safety for relatives of older adults, who experience reduced stress because the users are more independent [29, 59]. “With the use of security cameras at night and automatic medicine dispensers, the individual becomes less dependent on healthcare providers making visits to the home” [56, p. 28]. The Norwegian National Health and Hospital Plan claims that digital access to medical records strengthens patient safety, as it makes it possible for patients to see who has viewed information about them [52].

### Digital strategies and eHealth policies concerning older adults’ dignity

The three Scandinavian countries’ documents on digital strategies and eHealth policies concerning older adults’ dignity highlighted the importance of digital device security, user access to data and the human dimension of care.

#### Digital device security

All three Scandinavian countries emphasise the importance of security in their digital strategies and eHealth policies [8, 22, 51, 53, 63]. Companies, organisations and individuals should trust and be comfortable with the use of digital services [22, 29, 53, 63]. “The aim is that patients should experience the health system as a coherent and trustworthy health network for all that is both inherently digital and inherently personal” [22, p. 4]. Device security can help to reduce the barriers to the adoption of technological solutions [64, 69]. Included document dealing with technologies in care for older adults reveals that digital devices connected to the Internet with poor security may be vulnerable to hacking, which entails a risk to video and voice recordings, and the possibility of the device being controlled remotely by an attacker [29]. Surveyed documents from all the three Scandinavian countries suggest that attention to digital safety and the security of individuals is essential when exploiting the opportunities offered by new technologies and digital devices [8, 22, 29, 53, 69].

#### Access to data and the human dimension of care

Surveyed documents reveal a lack of coherence among healthcare sectors and digital systems in different regions, even in the same Scandinavian country [29, 61, 62, 64]. In Norway and Sweden, two sets of patient-accessible electronic health records are available, and the data cannot be transferred between those two services in one country. Patients receiving care in different regions therefore need to use several systems to access their data in its entirety [62, 64]. In contrast, Denmark uses a one-service-one-login approach and aims to make data available for everyone involved in a treatment [17, 62, 65]. Included documents add that there is a lack of digital competence among older adults and patients may have problems using digital healthcare systems [40, 52, 64].

Furthermore, the documents reviewed for this study claim that technology cannot replace the human dimension of care [29, 52, 54, 68]. Digital solutions are not always the best, especially if they risk replacing all face-to-face contacts with digital solutions, this can have negative effects on levels of social inclusion and human interaction [29, 52, 54, 68]. “For many patients and many types of examinations, it is less relevant to replace in-person meetings with digital solutions” [52, p. 95]. Reduced social stimulus could lead to person`s need for human contact not be met, and thereby affect human dignity in a negative way.

## Discussion

This study reviewed documents describing national digital strategies and eHealth policies in three Scandinavian countries. The purpose was to provide insights relevant to research questions about digital strategies and eHealth policies concerning older adults’ dignity in three Scandinavian countries: Norway, Sweden and Denmark.

The findings concerning older adults’ unequal access to digital services are in accordance with European studies from the last five years that also indicate a health and age-related digital divide [70, 71]. Such findings do not appear to evidence the impact of the principle of equality that informs the Nordic welfare model [13] and the UN sustainability goals for ensuring equal opportunities for all [72]. All three Scandinavian countries have programs designed to support and increase the digital literacy of older adults. This is important, as research suggests that older adults need educational support to be included in the digital society [73, 74]. Nevertheless, a recent study claims that Norwegian older adults experience that there are expectations towards them to have digital skills that they struggle to achieve and that affects their experience of dignity [34]. Our document analysis revealed many national policies make claims that programmes aiming to support and increase digital competence will help older adults to better adapt to the digital environment. The Scandinavian countries supportive programs has the European Commission’s aim for shaping Europe’s digital future for every citizen to benefit from digitised society, however this strategy needs local policies and collaboration with end-users to fully success [75]. Good practice of care involves ensuring people always feel valued when using healthcare services and that they are treated with respect, dignity and compassion [76]. National eHealth policy impacts the users of digital systems. The need to ask for help when trying to use eHealth systems may make older adults feel more vulnerable, and this can in turn, affect their experience of dignity, as dignity is in its variations a gathering of both common values and vulnerability [4, 34]. Dignity can be lost through vulnerability, and the need to ask for help may impact an older adult’s dignity in a negative way.

Another important challenge when using digital technology in healthcare systems is the human dimension of care. This includes dimensions where a person experiences that they feel human in the interaction with technology. Our findings on this issue are in line with the Code of Ethics for Nurses, which state that it is vital to make sure that technological devices do not replace human relationships [77]. The results indicate about policies considering that digital healthcare may be too easily substituted for in-person face-to-face contact, and this in turn can have negative effects on social inclusion. Social connections are essential for mental and physical health and well-being and these considerations support Scandinavian ageing-related policies for each individual to be supported to remain in good physical and mental health for as long as possible [10]. This is in accordance with the 3^rd^ UN sustainability goal to ensure healthy lives and promote well-being for all at all ages [72]. As eHealth has the potential to misrepresent or incompletely represent the human aspect of medical communication [78], we acknowledge the importance of discussions of this issue in the eHealth policies of the Scandinavian countries. A person has a need for human contact, there is a risk it not being met if social stimulus is reduced. This may lead to suffering among older adults by affecting their sense of dignity. Person`s sense of dignity can be promoted through human relationships, social inclusion and positive relationships with healthcare providers [79].

The results of this study show that the reviewed documents underline the importance of security in national digital strategies and eHealth systems. Our findings offer an overview of eHealth policies consequences for the user; eHealth systems that are vulnerable to hacking may make users insecure. The issue of trust in digital technology and eHealth systems has been recent topic of discussion in Scandinavian countries. Older people in Sweden have had problems trusting the eHealth tool because it has not always worked properly [80]. Older adults in Norway have found that they cannot always rely on eHealth systems, as they lack information about how the systems are used in healthcare and who has access to their personal data [34]. Such feelings of insecurity when using national eHealth systems may impact older adults’ dignity. Systems that are capable of processing personal data will be subjected to regulation under the EU General Data Protection Regulation, which requires data protection safeguards to be built into technology early in the development process and helps users to increase their trust in technology [81]. Our findings elucidate the importance of digital security in national digital strategies and eHealth policies, whereby feelings of security may affect older adults’ dignity in a positive way.

This study shows how national eHealth policies in three Scandinavian countries aim to give patients more opportunities to participate in their own healthcare. This is in accordance with World Health Organization’s claim that eHealth can be used to increase the level at which patients engage with their care [82]. The policies thereby underline the centrality of individuals’ conditions and needs when implementing new technologies in healthcare. This is in line with Foster and Sethares’s [83] claim that it is important to keep the patient’s perspective at the forefront if we want older adults to adopt eHealth systems. The findings of this study also reveal, how including older people in the process may influence policy-making and care. Engagement between research and policy is driven by systematic factors [84]. Nevertheless, the results do not reveal practical steps for achieving this goal, including what kind of regulatory regimes should apply to corporate service providers or which ministries are best placed to have responsibility for these issues. Reviews from the last decade show that the true needs of older people as end-users have been poorly understood when ensuring that digital technologies and eHealth systems meet their needs [85–88]. The inclusion of older adults’ voices and needs during the implementation of eHealth systems may impact their experience of dignity in a positive way, as dignity is the affirmation of something valuable in oneself or another [4].

On the other hand, giving older adults a voice is not a complete solution when implementing new technologies and improving healthcare, as health promotion is about more than just offering more choices [89]. Sometimes patients are necessarily rendered passive due to their situation, or health condition and technological systems have to be able balance service user agency and the new demands for agency placed on the older person by technology itself. In other words, a balance has to be struck between meeting the older person in their needs and making use of technology to facilitate, but an instrumental shift to technology as an *either* technology *or* human contact is not sufficient and can add to the problems technology is trying to solve. While putting the patient at the centre of the care contributes to a wider range of choices, choice alone does not meaningfully address well-being resources and absence of well-being needs [90]. In healthcare policy, making a patient’s autonomy too pervasive may also affect their dignity [89]. If it becomes too dominant that patients should be their own masters, then it may risk obstructions to the help the patient needs. eHealth systems offer more and more empowerment, but they may not be the full solution as patients’ deeper existential issues must also be taken care of [90]. In addition, technology can inadvertently marginalise older adults. This is in accordance with the studied strategies and policies and their focus on the importance of equal access to healthcare services, and the 10^th^ UN sustainability goal to reduce inequalities both within and among countries [72].

### Strengths and limitations

It is a strength of this study that papers in all three Scandinavian languages and in English were considered for inclusion. While the authors include native speakers of Danish, Norwegian and English and four can read and/or speak Swedish, our collective skills in the latter are less developed, hence there was special attention paid to the documents in Swedish to capture all the relevant data. The use of a comprehensive, systematic search strategy and including documents according to the JBI framework [45] in this study can also be seen as a strength. It provided transparency to this process for the authors and is thereby considered as a strength. We added six analytic steps suggested by O`Leary to secure further analytical depth to the study [43].

The reviwed documents provided background information that helped us to understand the roots of specific issues and indicated the conditions that influence the phenomena under investigation [38]. Although documents can be a rich source of data, researchers should examine documents with a critical eye [38]; thus, it is a strength of this study that the included texts were not only reviewed by three of the authors but also critically reviewed using the JBI Critical Appraisal Checklist for Text and Opinion Papers [49]. Furthermore, this study offers new insights into digital strategies and eHealth policies concerning older adults’ dignity in three Scandinavian countries. However, corresponding limitations include not seeking to differentiate the three countries’ digital strategies and eHealth policies concerning older adults’ dignity, which could be further research and the challenges presented by document analysis as a research method. For example, non-academic documents are produced for purposes other than research and do not reflect a research agenda, meaning that they do not always provide sufficient detail [38]. Although document analysis is often combined with other qualitative research methods [38], this study used only one method. Nonetheless, the findings of this document analysis add to the available evidence about the three Scandinavian countries’ national digital strategies and innovative eHealth policies with aspects concerning older adults’ dignity.

## Conclusions

This document analysis presents the three Scandinavian countries’ national digital strategies and innovative eHealth policies concerning older adults’ dignity. All three countries in this study underline the importance of security in their digital services. The documents we reviewed describe a lack of digital competence among older adults. Support for digital competence is needed, otherwise older adults may encounter increasing marginalisation, loss of agency, and perceived stereotypically as ‘a problem group’ when using digital healthcare systems. There is a risk that the need to ask for help to use eHealth systems may cause suffering among older adults. This complex issue may affect their experience of sense of their personal dignity, of their affiliation to society. It is time to increase our understanding of human dignity in this arena and focus on older adults’ needs as ‘end-users’ if we want them to gain from digital solutions and eHealth systems. On the other hand, patient empowerment in this arena and the use of eHealth systems alone cannot be the full solution to safeguarding older adults` dignity. New digital services must be meaningfully integrated into countries’ digital strategies and eHealth policies, which requires investigation that goes beyond as ‘end-user’ experiences of technology to provide an understanding of how we can support human dignity through technology—an area that has so far received little attention. To the extent the three Scandinavian countries national healthcare strategies and policies for digital development and eHealth have innovative power in relation to the dignity of older adults**,** the most clearly are that they all emphasize the importance of equal access to healthcare services. With that, the 10^th^ UN sustainability goal to reduce inequality is followed, which states a powerful argument for national and local policy making. As thus they promote a stance of dignified care.

## Data Availability

All data generated or analysed during this study are included in this published article.

## Abbreviations

WHO: World Health Organization
JBI: Joanna Briggs Institute
UN: United Nations
